# Summary of best evidence for drainage tube management to prevent surgical site infections

**DOI:** 10.1186/s12893-026-03962-5

**Published:** 2026-07-02

**Authors:** Qingqing Du, Changpei Chen, Shan Wu, Fangyuan Sun, Hailei Bian, Li Ni

**Affiliations:** 1 Shanghai Children’s Hospital, School of Medicine, Shanghai Jiao Tong University, Shanghai, 200062 China; 2 Tongji University School of Medicine, Shanghai, 200092 China; 3 Shanghai East Hospital, School of Medicine, Tongji University, Shanghai, 200120 China

**Keywords:** Surgical Site Infection (SSI), Drainage Tube, Drainage Tube Management, Infection Prevention, Evidence Summary, Evidence-Based Nursing

## Abstract

**Objective:**

To conduct a narrative evidence synthesis integrating clinical practice guidelines, systematic reviews, meta-analyses, expert consensus, and primary studies to summarize the best available evidence related to drainage tube management for the prevention of surgical site infections (SSI), and to develop an evidence-based framework for clinical standardized management strategies.

**Methods:**

Utilizing the “6S” evidence model, a systematic search was conducted on various national and international databases and websites up to October 31, 2025, including WHO, CDC, NICE, SHEA/IDSA, APIC, GIN, Medlive Guideline Network, The Cochrane Library, Joanna Briggs Institute (JBI) Evidence Synthesis, PubMed, Web of Science, Embase, CINAHL, ClinicalTrials.gov, CBM, CNKI, VIP, and WanFang. The search aimed to extract relevant evidence regarding the management of drainage tubes to prevent SSI. Two researchers independently performed the literature screening, quality assessment, evidence extraction, and integration of the literature.

**Results:**

A total of 21 publications were included, comprising 4 guidelines, 4 expert consensuses and opinions, 2 evidence summaries, 6 systematic reviews, 3 randomized controlled trials, and 2 cohort studies. A total of 40 pieces of best evidence were summarized, covering ten aspects: indications and selection for drainage tube placement, intraoperative principles of drainage tube placement, daily maintenance of drainage tubes, management of drainage tube blockage, infection prevention measures, application of antimicrobial drugs, timing for drainage tube removal, monitoring and assessment of drainage fluid, patient health education, and referral indications.

**Conclusion:**

The best evidence summary for the management of drainage tubes to prevent SSI provides a preliminary evidence-based reference for the standardized management practice of drainage tubes in clinical settings. Clinicians and nursing staff should integrate specific circumstances and professional judgment to translate evidence into practice, thus providing scientific management and guidance for patients with drainage tubes.

**Supplementary Information:**

The online version contains supplementary material available at https://doi.org/10.1186/s12893-026-03962-5.

## Introduction

Surgical Site Infection (SSI) refers to the infection occurring at the surgical incision site, accounting for approximately 15% to 20% of all hospital-acquired infections [1]. According to the Centers for Disease Control and Prevention, approximately 160,000 to 300,000 patients in the US develop SSI annually, resulting in prolonged hospital stays and increased medical costs [2]. The occurrence of SSI causes physical and emotional pain to patients and significantly increases medical resource consumption [3].

A drainage tube is a commonly used auxiliary device after surgical operations to remove fluids, blood, or exudates from the surgical site, preventing infections caused by fluid accumulation [4]. Since British surgeon Lawson Tait introduced the concept of “When in doubt, place a drain” in the 19th century, postoperative prophylactic drainage tube placement has become routine [5]. However, recent studies have shown that drainage tubes themselves can become a pathway for bacterial colonization, and improper management may increase SSI risk [6, 7].

With the widespread adoption of Enhanced Recovery After Surgery (ERAS) protocols, management strategies for drainage tubes are continually being updated [8]. The World Health Organization’s 2018 “Global Guidelines for the Prevention of SSI” states that drainage tube presence should not justify prolonged perioperative antimicrobial prophylaxis, and tubes should be removed as soon as clinically permissible [9]. In 2023, SHEA/IDSA/APIC updated their SSI prevention strategies, underscoring the importance of standardized drainage tube management [10]. In China, the Surgical Branch of the Chinese Medical Association released SSI prevention guidelines in 2019 [11] and ERAS practice guidelines in 2021 [12]. However, a systematic summary of evidence regarding surgical drainage tube management remains scarce. This study aims to conduct a narrative evidence synthesis integrating guidelines, systematic reviews, and primary studies to summarize the best available evidence for drainage tube management in SSI prevention, translating existing high-quality evidence into actionable clinical guidance.

## Materials and methods

### Establishment of the research question

In accordance with the PIPOST model, the research question for evidence-based practice was established as follows: The target population (P) includes patients with postoperative drainage tubes; the intervention (I) entails the management of drainage tubes, which includes their placement, maintenance, monitoring, and removal; the professionals (P) involved are clinical medical and nursing staff; the outcome measures (O) include the incidence of SSI, complications related to drainage tubes, and length of hospital stay; the settings (S) for clinical evidence application include hospitals, communities, and home care; and the types of evidence (T) considered are clinical decision-making tools, guidelines, systematic reviews, evidence summaries, expert consensus, and relevant primary research.

### Literature search strategy

#### Search resources

Guided by the 6 S model of evidence hierarchy, a sequential search was conducted as follows: Evidence-based databases including The Cochrane Library and JBI Evidence Synthesis were searched. Websites of international organizations and guidelines such as the World Health Organization (WHO), Centers for Disease Control and Prevention (CDC), UK’s National Institute for Health and Care Excellence (NICE), Society for Healthcare Epidemiology of America/Infectious Diseases Society of America (SHEA/IDSA), Association for Professionals in Infection Control and Epidemiology (APIC), Guidelines International Network (GIN), Surgical Infection Society (SIS), and Medlive Guideline Network were consulted. English-language databases searched included PubMed, Web of Science, Embase, CINAHL, and ClinicalTrials.gov. Chinese-language databases included China Biomedical Database (CBM), China National Knowledge Infrastructure (CNKI), VIP, and WanFang. EndNote 21 (Clarivate Analytics) was used for reference management and duplicate removal.

#### Search strategy

English keywords: surgical site infection, SSI, surgical drain, drain management, drain care, infection prevention, drain removal, aseptic technique, wound drainage. The final search across all databases was conducted on October 31, 2025, covering publications from inception to this date.

The search strategy was constructed using a combination of subject headings (MeSH for PubMed, Emtree for Embase) and free-text keywords, connected with Boolean operators (AND, OR). The search string for PubMed was designed as follows:

#1 “Surgical Wound Infection“[Mesh] OR “Surgical Site Infection“[Title/Abstract] OR “SSI“[Title/Abstract] OR “Postoperative Infection“[Title/Abstract] OR “Wound Infection, Surgical“[Title/Abstract].

#2 “Drainage“[Mesh] OR “Surgical Drain“[Title/Abstract] OR “Drain Management“[Title/Abstract] OR “Drain Care“[Title/Abstract] OR “Wound Drainage“[Title/Abstract] OR “Postoperative Drain“[Title/Abstract] OR “Drain Removal“[Title/Abstract] OR “Suction Drain“[Title/Abstract] OR “Closed Suction Drain“[Title/Abstract].

#3 “Infection Prevention“[Title/Abstract] OR “Infection Control“[Mesh] OR “Aseptic Technique“[Title/Abstract] OR “Antibiotic Prophylaxis“[Mesh] OR “Negative Pressure Wound Therapy“[Mesh].

#4 #1 AND #2.

#5 #4 AND #3.

#6 #4 OR #5.

Filters: English, Chinese; Publication date: from inception to October 31, 2025.

Similar search strategies were adapted for Embase (using Emtree terms), Web of Science, CINAHL, and the Cochrane Library. For Chinese databases (CBM, CNKI, VIP, WanFang), equivalent Chinese search terms were used, including the Chinese translations of the following English terms: surgical site infection, drainage tube, drainage tube management, infection prevention, and drain removal.

### Criteria for inclusion and exclusion of literature

#### Inclusion criteria

Publications such as guidelines, statements, expert consensus or opinions, evidence summaries, systematic reviews, and randomized controlled trials (RCTs) that address the management and intervention of drainage tubes to prevent SSI in both English and Chinese literature.

Specifically, eligible studies met the following criteria: (1) Population: adult or pediatric patients undergoing surgical procedures with postoperative drainage tube placement; (2) Intervention: any aspect of drainage tube management, including placement techniques, maintenance care, monitoring protocols, and removal strategies; (3) Comparator: standard care, alternative drainage management strategies, or no drainage; (4) Outcome: primary outcomes included SSI incidence (defined by CDC/NHSN criteria or similar standardized definitions); secondary outcomes included drainage-related complications, length of hospital stay, and patient-reported outcomes; (5) Setting: acute care hospitals, ambulatory surgical centers, or post-discharge home care; (6) Evidence type: clinical practice guidelines, expert consensus statements, evidence summaries, systematic reviews, meta-analyses, RCTs, and prospective cohort studies.

#### Exclusion criteria

Incomplete guidelines or those comprising only introductions, tables of contents, abstracts, or brief guides, as well as outdated versions of recently revised guidelines; non-English and non-Chinese literature; studies with poor quality assessments; and literature for which full texts are unavailable.

Additional exclusion criteria included: (1) studies focusing exclusively on drainage tube management for non-infectious purposes (e.g., cancer chemotherapy drainage, pleural effusion drainage without surgical context); (2) case reports, case series with fewer than 10 patients, editorials, and letters without original data; (3) studies published only as conference abstracts without subsequent full-text publication; (4) studies with critical methodological flaws identified during quality appraisal (e.g., inappropriate statistical methods, high risk of bias); (5) duplicate publications or overlapping study populations (only the most recent or comprehensive publication was retained); (6) animal studies or in vitro experiments.

### Criteria for quality assessment of literature

In accordance with the diverse types of literature, corresponding quality assessment tools are employed. The guidelines utilize the Appraisal of Guidelines for Research & Evaluation II (AGREE II) [13] for achieving consensus and gathering expert opinions on quality assessments. For articles based on expert opinion and professional consensus, the JBI Critical Appraisal Checklist for Text and Opinion (2016) is applied [14]. For evidence summaries, the quality is assessed using the Critical Appraisal for Summaries of Evidence (CASE) tool [15]. Systematic reviews are evaluated with the JBI Critical Appraisal Checklist for Systematic Reviews (2016), whereas RCTs are assessed using the JBI Critical Appraisal Checklist for Randomized Controlled Trials (2016). Cohort studies are evaluated through the JBI Critical Appraisal Checklist for Cohort Studies (2016) [14].

Two researchers independently conducted all quality appraisals. For AGREE II, each of the six domains was scored on a 7-point Likert scale (1 = strongly disagree to 7 = strongly agree), and standardized domain scores were calculated as: (obtained score - minimum possible score) / (maximum possible score - minimum possible score) x 100%. Studies with standardized domain scores of ≥ 60% in at least five of six domains were considered high quality and eligible for inclusion. For JBI checklists, studies were rated as “Include” (all criteria met or only minor concerns), “Exclude” (major methodological flaws), or “Seek further info” (insufficient information to assess). Discrepancies between the two reviewers were resolved through discussion and consensus; when consensus could not be reached, a third senior reviewer (L.N.) was consulted to make the final determination.

### Management of evidence overlap and double-counting

Given that this review included systematic reviews, guidelines, and primary studies, potential overlap of evidence was carefully managed. For systematic reviews, we extracted only the highest-level evidence (e.g., meta-analysis results rather than individual RCT findings) to avoid double-counting primary studies. When a primary study was included in multiple systematic reviews, the study was counted only once in the evidence synthesis, and the systematic review providing the most comprehensive analysis was prioritized. Guidelines and expert consensus were treated as independent sources of recommendations, but when they cited the same underlying primary studies, the evidence was mapped back to the original source to ensure traceability.

#### Assessment of overlapping evidence and reconciliation of conflicting recommendations

To ensure evidence traceability and minimize double-counting, the following procedures were applied:

(A) Overlap assessment: All included systematic reviews were screened for overlapping primary studies. When the same RCT or cohort study appeared in multiple systematic reviews, it was counted only once in the evidence synthesis, and the systematic review providing the most comprehensive meta-analysis was prioritized.
(B) Double-counting prevention: Primary studies already incorporated into included systematic reviews were not counted separately in the evidence grading. The evidence level was assigned based on the highest-quality synthesis available.
(C) Conflicting recommendations: When discrepancies existed between systematic reviews and primary studies, the most recent and methodologically rigorous source was prioritized. When guidelines provided conflicting recommendations, consistency across multiple high-quality sources was assessed, and the recommendation supported by the strongest direct evidence was adopted. Any downgrades due to indirect evidence were explicitly noted.

### Data extraction, integration, and grading of evidence

#### Data extraction

The literature screening, quality evaluation and evidence grading were independently conducted by two researchers who had received training in evidence-based nursing.

Discrepancies between the two reviewers were resolved through discussion and consensus; when consensus could not be reached, a third senior reviewer was consulted to make the final determination.

EndNote 21 (Clarivate Analytics) was used for citation management and duplicate removal.

Grey literature was not systematically searched; only published guidelines, systematic reviews, and primary studies from the specified databases and guideline websites were included.

#### Evidence grading and recommendation levels

The preliminary grading of evidence was performed using the JBI Evidence-Based Healthcare Center’s intervention research evidence pre-grading system (2014 edition) [16]. Following the preliminary grading, the recommendation strength of the evidence was determined under the guidance of the FAME framework: Grade A: strongly recommended; Grade B: weakly recommended.

The evidence-grading process followed a hierarchical approach: Level 1a evidence was assigned to recommendations derived from high-quality systematic reviews or meta-analyses; Level 1b was assigned to recommendations supported by individual high-quality RCTs or well-designed cohort studies; Level 1c was assigned to recommendations based on expert consensus or guideline recommendations where direct comparative evidence was limited. For each of the 40 evidence statements, the specific source(s) supporting the recommendation were documented, and the mapping between evidence level and underlying study types is transparently reported in Table 6. Where indirect evidence was used (e.g., extrapolating from general SSI prevention principles to drainage tube management), this was explicitly noted and the recommendation strength was downgraded accordingly from Grade A to Grade B.

## Results

### Literature retrieval results

**Fig. 1:**
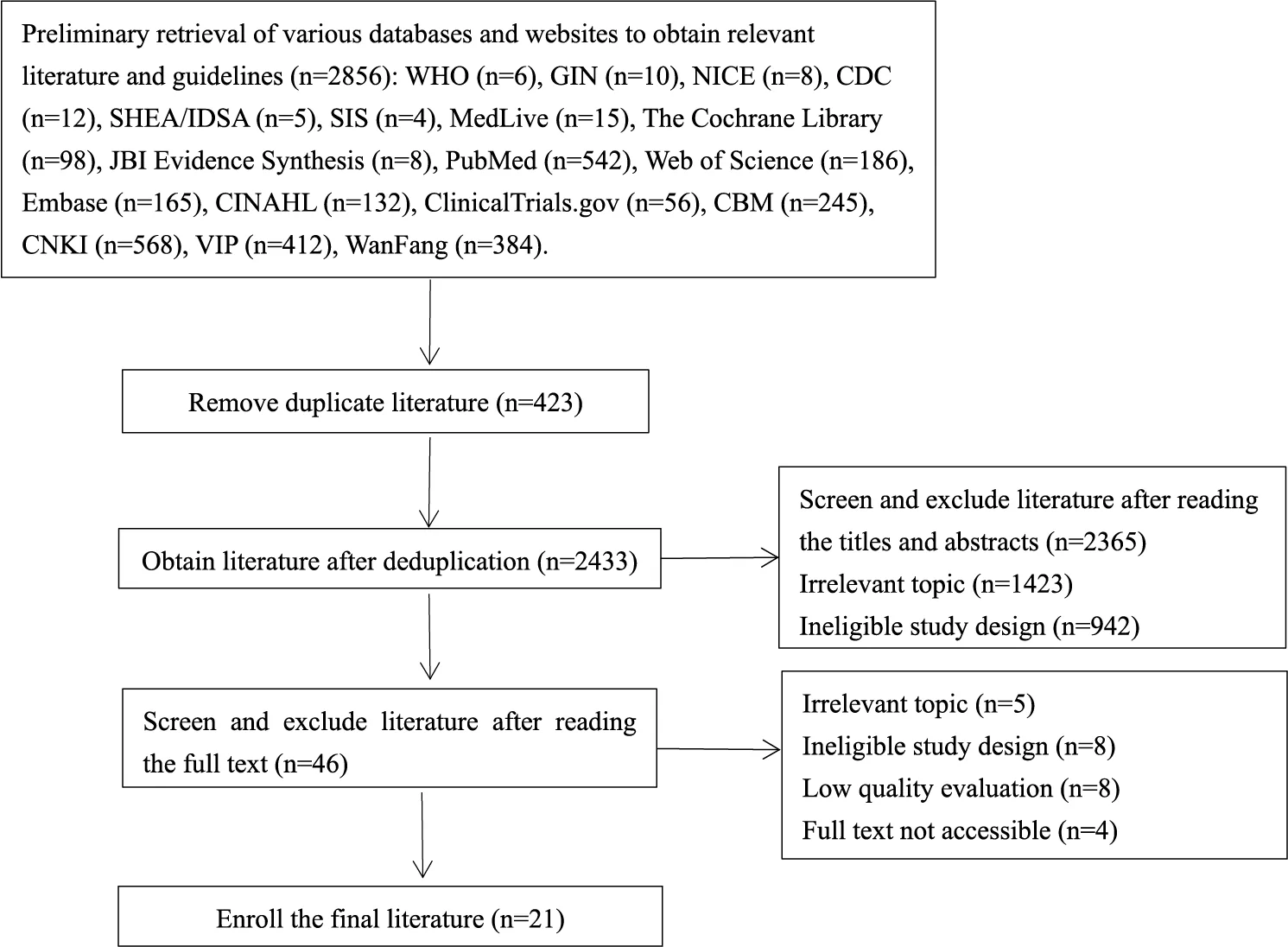
Literature screening process

A total of 21 studies were included, comprising 4 guidelines, 4 expert consensuses and opinions, 2 evidence summaries, 6 systematic reviews, 3 RCTs, and 2 cohort studies. The literature screening process followed PRISMA 2020 reporting guidelines. The process of literature selection is depicted in Fig. 1, and the basic information of the included studies is presented in Table 1.

**Table 1:** Basic information of the enrolled literature (*n* = 21)

Number	Literature name	Year	Country/Institution	Topic	Source/Publishing organization	Evidence type
G1 [10]	Strategies to prevent surgical site infections in acute-care hospitals: 2022 Update	2023	US	SSI prevention strategies	SHEA/IDSA/APIC	Guideline
G2 [17]	Surgical Site Infections: Prevention and Treatment (NG125 Update)	Updated 2024	US	SSI prevention and treatment	NICE	Guideline
G3 [18]	The Surgical Infection Society Guidelines on the Management of Intra-Abdominal Infection: 2024 Update	2024	US	Management of Intra-abdominal Infection	SIS	Guideline
G4 [19]	The American Society of Colon and Rectal Surgeons Clinical Practice Guidelines for Preventing Surgical Site Infection	2024	US	SSI prevention	ASCRS	Guideline
C1 [11]	Chinese Guidelines for the Prevention of Surgical Site Infections	2019	PRC	SSI prevention and drain management	Chinese Society of Surgery, Chinese Medical Association	Expert consensus
C2 [12]	Clinical Practice Guidelines for Enhanced Recovery after Surgery in China (2021)	2021	PRC	ERAS and drain management	Chinese Society of Surgery and Anesthesiology, Chinese Medical Association	Expert consensus
C3 [8]	Consensus statement for perioperative care in lumbar spinal fusion: ERAS Society recommendations	2021	International	ERAS and drain management	ERAS Society	Expert consensus
C4 [20]	ERAS Cardiac Society turnkey order set for SSI prevention	2024	International	SSI prevention in cardiac surgery	ERAS Cardiac Society	Expert opinions
E1 [21]	Reflections on prevention and management strategies for surgical site infections	2025	PRC	SSI prevention management	Guo YJ et al.	Evidence summary
E2 [22]	Evidence analysis for surgical site infection prevention in adult inpatients based on guidelines and clinical decision-making	2024	PRC	Postoperative infection prevention	Lei QM et al.	Evidence summary
SR1 [23]	Optimizing surgical drain removal: a narrative review of timing, criteria, and evidence-based practices	2025	International	Timing for drain removal	Cureus	Systematic review
SR2 [24]	An optimal timing for removing a drain after breast surgery: a systematic review and meta-analysis	2021	Japan	Drains in breast surgery	Shima H et al.	Systematic review
SR3 [25]	Recent evidence for subcutaneous drains to prevent surgical site infections after abdominal surgery: A systematic review and meta-analysis	2023	International	Subcutaneous drain	World J Gastrointest Surg	Systematic review
SR4 [26]	Update on the management of surgical site infections	2022	Italy	SSI management	Antibiotics	Systematic review
SR5 [27]	Care bundles in surgical site infection prevention: a narrative review	2024	International	Care bundles	Curr Infect Dis Rep	Systematic review
SR6 [28]	Intraoperative wound irrigation to prevent surgical site infection: a systematic review and meta-analysis	2024	International	Intraoperative wound irrigation	World J Surg	Systematic review
R1 [29]	Early versus delayed drain removal after abdominal surgery: a multicenter RCT	2022	International	Drains in abdominal surgery	Multicenter study	RCT
R2 [30]	Negative Pressure Wound Therapy for the Prevention of Surgical Site Infections Using Fascia Closure After EVAR—A Randomized Trial	2022	Sweden	Prevention of SSI with NPWT after EVAR	World J Surg	RCT
R3 [31]	Prevention of incisional surgical site infection by subfascial closed suction drainage after open laparotomy	2023	Japan	Subfascial drainage in open abdominal surgery	Patient Saf Surg	RCT
CS1 [32]	Adverse events of surgical drain placement: an analysis of the NSQIP database	2024	US	Drainage tubes and postoperative complications	Talwar A et al.	Cohort study
CS2 [6]	Influence of Drain Characteristics and Other Known Risk Factors on Surgical Site Infection Occurrence in Plastic Surgery Patients	2023	Korea	Drainage tube characteristics and SSI	Na YC et al.	Cohort study

### Evaluation of literature quality

#### Guideline quality assessment

The AGREE II quality assessment of the four guidelines indicated that the highest standardized percentage (%) areas were “Scope and Purpose” and “Clarity”; the lower scoring areas were “Stakeholder Involvement” and “Applicability.” The percentages represent standardized domain scores calculated as (obtained score - minimum possible score) / (maximum possible score - minimum possible score) x 100%, where each domain is rated on a 7-point Likert scale (1 = strongly disagree to 7 = strongly agree) by two independent appraisers. All guidelines had more than five domains scoring ≥ 60%, indicating a generally high overall quality (Table 2).

**Table 2:** Quality assessment of guidelines (*n* = 4)

Number	Scope and Purpose (%)	Stakeholder Involvement (%)	Rigor of Development (%)	Clarity of Presentation (%)	Applicability (%)	Editorial Independence (%)
G1	92	78	85	88	72	83
G2	89	72	82	85	68	80
G3	86	68	78	83	65	78
G4	84	65	75	80	62	75

#### Quality evaluation of expert consensus and opinions

The JBI quality assessment results for four articles on expert consensus and opinions (C1–C4) indicated that all documents explicitly cited the sources of the opinions, which were based on analyses conducted by influential experts in the field and were centered around the interests of the study population. The expression of opinions is logical and references existing literature. Specifically, C1, C3, and C4 met all six criteria (clear source of opinions, opinions based on analysis, derived from influential experts, centered on relevant population interests, logical presentation, and consistency with previous literature). C2 met five of six criteria, scoring “No” on consistency with previous literature due to limited citation of published evidence, but was retained given its comprehensive coverage of ERAS principles.

#### Quality evaluation of evidence summaries

The two evidence summaries that were evaluated (E1 and E2) both demonstrated high overall quality according to the Critical Appraisal for Summaries of Evidence (CASE) tool. Both documents met all ten evaluation criteria: specific scope and subjects, clarity and transparency of authorship, clarity and transparency of the review process, transparency and comprehensiveness of the search, clear grading of evidence, clarity of recommendations, appropriateness of citation in recommendations, timeliness of recommendations, declaration of conflicts of interest, and relevance to the study population. Both evidence summaries trace back to original documents and assess them, and are deemed suitable for inclusion.

#### Quality evaluation of systematic reviews

The JBI quality assessment results for six systematic reviews reveal that all documents clearly articulate the evidence-based questions, with appropriate criteria for inclusion, search strategies, literature quality evaluations, and methods for combining studies. However, some documents did not have sufficient search resources and did not assess the potential for publication bias (Table 3).

**Table 3:** Quality evaluation of systematic reviews (*n* = 6)

Number	Q1	Q2	Q3	Q4	Q5	Q6	Q7	Q8	Q9	Q10	Q11
SR1	Yes	Yes	Yes	Yes	Yes	Yes	Yes	Yes	Yes	Yes	Yes
SR2	Yes	Yes	Yes	Yes	Yes	Yes	Yes	Yes	Yes	Yes	Yes
SR3	Yes	Yes	Yes	Yes	Yes	Yes	Yes	Yes	Unclear	Yes	Yes
SR4	Yes	Yes	Yes	Yes	Yes	Yes	Yes	Yes	Yes	Yes	Yes
SR5	Yes	Yes	Yes	Yes	Yes	Yes	Yes	Yes	Yes	Yes	Yes
SR6	Yes	Yes	Yes	Yes	Yes	Yes	No	Yes	Yes	Yes	Unclear

Q1: Clarity of the posed question; Q2: Appropriateness of inclusion criteria; Q3: Appropriateness of the search strategy; Q4: Sufficiency of search resources; Q5: Appropriateness of literature quality evaluation criteria; Q6: Evaluation conducted by two or more persons; Q7: Appropriateness of methods for combining studies; Q8: Assessment of publication bias; Q9: Conclusions supported by analysis; Q10: Comprehensive discussion of heterogeneity; Q11: Appropriateness of recommendations

#### Quality assessment of RCT

The JBI quality assessment results of three RCTs indicate that all included studies demonstrated comparability of baseline characteristics between groups, uniformity in outcome measurement methods, and appropriateness of data analysis techniques. However, some studies did not adequately report on allocation concealment and blinding procedures (Table 4).

**Table 4:** Quality assessment of RCT (*n* = 3)

Number	Q1	Q2	Q3	Q4	Q5	Q6	Q7	Q8	Q9	Q10	Q11	Q12	Q13
R1	Yes	Yes	Yes	Yes	Yes	Yes	Yes	Yes	Yes	Yes	Yes	Yes	Yes
R2	Yes	Unclear	Yes	Unclear	Unclear	Yes	Yes	Yes	Yes	Yes	Yes	Yes	Yes
R3	Yes	Yes	Yes	Yes	Unclear	Yes	Yes	Yes	Yes	Yes	Yes	Yes	Yes

Q1: True randomization of group assignment; Q2: Concealment of allocation; Q3: Comparability of baseline characteristics between groups; Q4: Blinding of participants; Q5: Blinding of intervention providers; Q6: Blinding of outcome assessors; Q7: Uniformity of interventions across groups; Q8: Complete follow-up; Q9: Intention-to-treat analysis; Q10: Uniformity of outcome assessment methods across groups; Q11: Reliability of outcome assessment methods; Q12: Appropriateness of data analysis techniques; Q13: Appropriateness of trial design

#### Quality assessment of cohort studies

The JBI quality assessment results for two cohort studies show that the studies were well-designed, with accurate measurement of exposure factors, adequate follow-up, and appropriate statistical analysis, indicating a high overall quality (Table 5).

**Table 5:** Quality assessment of cohort studies (*n* = 2)

Number	Q1	Q2	Q3	Q4	Q5	Q6	Q7	Q8	Q9	Q10	Q11
CS1	Yes	Yes	Yes	Yes	Yes	Yes	Yes	Yes	Yes	Yes	Yes
CS2	Yes	Yes	Yes	Yes	Yes	Yes	Yes	Yes	Yes	Yes	Yes

Q1: Both groups are from the same population; Q2: Accurate measurement of exposure factors; Q3: Identification and management of confounding factors; Q4: Absence of outcome at the time of exposure; Q5: Blinding of outcome assessors; Q6: Adequate follow-up; Q7: Reasonable loss to follow-up rate; Q8: Analysis of reasons for loss to follow-up; Q9: Accurate outcome measurement; Q10: Appropriateness of statistical analysis; Q11: Conclusions supported by analysis

### Summary, grading, and recommendations of evidence-based evidence

An analysis of the included literature covered ten aspects concerning the use of drainage tubes: indications and selection for placement, principles of intraoperative placement, routine maintenance, handling of blockages, infection prevention measures, application of antimicrobial drugs, timing of removal, monitoring and assessment of drainage fluid, patient health education, and referral indications, comprising 40 pieces of evidence. The evidence synthesis process integrated findings from all included source types (guidelines, systematic reviews, RCTs, and cohort studies) using a hierarchical approach. For each evidence statement, the primary supporting source was identified, and where multiple sources addressed the same recommendation, consistency across sources was assessed. When discrepancies existed between systematic reviews and primary studies, the most recent and methodologically rigorous source was prioritized. All 40 evidence statements, their corresponding evidence grades, recommendation strengths, and supporting source mappings are presented in Table 6. Specific grading and recommendations are presented in Table 6.

**Table 6:** Summary results of the evidence summary

Item	Evidence content	Evidence grading	Strength of recommendation
1. Indications and selection of drain placement	1. The placement of drainage tubes should be based on clear clinical indications, including: prevention of postoperative fluid accumulation (blood, lymph, exudate), drainage of infection foci or abscesses, and monitoring of postoperative bleeding or anastomotic leaks [11, 12].	1a	A
	2. A closed negative pressure drainage system is preferred over an open system to reduce the risk of SSI [6].	1a	A
	3. The diameter of the tube should be chosen based on the expected amount of drainage, with a preference for silicone or polyurethane materials to minimize tissue reaction and bacterial adhesion [11].	1c	B
	4. The decision to place a drainage tube should be individualized based on the surgical type and patient risk factors (e.g., diabetes, obesity, immunosuppression), and anticipated fluid accumulation, rather than applied routinely for all procedures [10, 17].	1b	B
2. Intraoperative principles for drain placement	5. The placement of drainage tubes intraoperatively should strictly adhere to aseptic techniques, and the tubes should be carefully checked for any twisting, folding, or compression [10, 11, 18].	1a	A
	6. The position of the drainage tube should be precisely located at the lowest point of the surgical field or where fluid is most likely to accumulate, ensuring that the side holes are completely within the target drainage area to avoid deep tissue damage or superficial placement that impedes drainage [11, 18, 24].	1b	A
	7. Abdominal surgery: For hepatic and biliary surgeries, the tube is usually placed in the subhepatic or subdiaphragmatic spaces; for pancreatic surgery, it is placed adjacent to the pancreatic margin, avoiding direct contact with vascular remnants [11, 18].	1b	A
	8. Spinal surgery: The decision to place a drainage tube depends on the extent of the surgery and the volume of bleeding, avoiding routine prophylactic placement [8].	1b	B
	9. The drainage tube should be led out through a separate puncture point that is 2–3 cm away from the surgical incision. Avoid placing the drainage tube through the main incision to reduce the risk of incision infection and rupture [11].	1b	A
	10. Before closing the incision after the surgery, it is necessary to confirm that the drainage tube is in the correct position, the drainage is unobstructed, and it is properly connected to the drainage bag or negative pressure device. Record the type, specification, placement location and initial drainage condition of the drainage tube, and complete the handover [11, 12].	1c	B
	11. After the drainage tube is led out through the skin, it should be properly fixed. It is recommended to use non-absorbable sutures to fix the drainage tube to the skin. The sutures should be secure but not too tight to avoid compressing the drainage tube and causing narrowing of the lumen [11].	1b	A
3. Routine drain maintenance	12. The care of the drainage tube should use an aseptic non-touch technique, including strict hand hygiene before and after handling, maintaining a closed drainage system, and avoiding touching critical parts [17].	1a	A
	13. Skin care around the drainage tube: Assess the skin condition at the puncture site daily, observing for redness, exudation, and secretion. Cover with sterile gauze or a transparent semi-permeable membrane dressing, and replace the dressing promptly if it becomes damp, loose, or contaminated [10, 17].	1a	A
	14. Drainage bag management: Regularly empty the drainage fluid (usually every shift or when it’s 2/3 full), using a separate container for collection, avoiding contact between the drainage bag outlet and the collection container. The drainage bag should always be positioned below the drainage site to prevent backflow [10, 11].	1a	A
	15. Keep the drainage tube clear: Regularly check the drainage tube for twists, compression, or blockages, and assess the necessity of the tube’s placement daily [6, 32].	1b	A
4. Management of drain blockage	16. Blockages in the drainage tube can occur at the opening of the tube or anywhere along the tube, commonly due to blood clots, tissue fragments, and fibrin deposition [17, 21].	1b	A
	17. Squeezing method for clearing blockages: Hold the drainage tube near the skin with one hand, and with the thumb and forefinger of the other hand, pinch the tube and steadily slide towards the drainage bag to effectively clear clots and debris from inside the tube [11].	1c	B
	18. Flushing method: When the squeezing method fails to clear a blockage, a small amount of sterile saline can be used for flushing [28].	1a	B
	19. The flushing procedure should strictly follow aseptic techniques to avoid introducing contaminants into the surgical site. After flushing, confirm that the drainage is clear, and record the volume of flushing fluid and the backflow situation [10, 17].	1a	A
	20. If repeated interventions fail to restore patency, promptly report to a doctor to assess the need for replacement or removal of the drainage tube [21].	1b	A
5. Infection prevention measures	21. Performing hand hygiene is the most basic measure to prevent infections related to drainage tubes [10].	1a	A
	22. Assess the necessity of the drainage tube daily and remove it as soon as it is no longer needed to reduce the risk of SSI, prolonged inflammatory response, and wound complications [23, 32].	1a	A
	23. Within 48 h post-surgery, cover the puncture site of the drainage tube with sterile dressings and clean the wound with sterile saline [17].	1a	A
	24. The puncture site of the drainage tube can be disinfected with 2% chlorhexidine alcohol. For high-risk patients, consider using chlorhexidine-soaked dressings to cover the puncture site, which can reduce the risk of bacterial colonization [1, 2].	1b	A
6. Antibiotic use	25. In clean and clean-contaminated surgeries, the presence of a drainage tube should not extend the duration of postoperative prophylactic antibiotic use [10].	1a	A
	26. Prophylactic antibiotics should be administered within 60 min before skin incision [26].	1a	A
	27. If the patient shows signs of infection (fever, localized redness, warmth, pain, or purulent drainage), adjust the antibiotic treatment plan based on culture and sensitivity results after taking specimens, instead of empirically extending the use of preventive medication [10, 17].	1a	A
7. Timing for drain removal	28. Criteria-based removal of drainage tubes: Consider removing the drainage tube when the 24-hour drainage volume is less than 30–50 mL, and the drainage fluid shows no signs of infection, bile, or bleeding [23].	1a	B
	29. Time-based removal: For some surgeries, a fixed time point removal strategy can be adopted. Drainage tubes can be removed 3 to 5 days after breast surgery, 24 to 48 h after joint replacement surgery, and 3 to 5 days after abdominal surgery [12, 24].	1b	B
	30. Early removal (within 5 days after surgery) does not increase the risk of SSI, while delayed removal (5 to 7 days) may increase the risk of SSI and prolong the hospital stay [24, 32].	1a	A
	31. Removal of drainage tubes under special circumstances should be individualized. After pancreatic surgery, monitoring the amylase level in the drainage fluid (< 600 U/L indicates safe removal), and after hepatobiliary surgery, monitoring bilirubin in the drainage fluid can guide removal decisions [18].	1b	B
8. Drainage fluid monitoring and assessment	32. Daily documentation of the volume, color, consistency, and odor of the drainage fluid is required. Normal postoperative drainage fluid typically transitions from sanguineous to serosanguineous to serous, gradually decreasing in volume. Any abnormal changes should be promptly reported and addressed [10].	1a	A
	33. Signs and management of abnormal drainage fluid: Purulent drainage indicates infection and requires culture; bile-like drainage suggests a bile leak; persistently increasing bloody drainage suggests bleeding, all requiring urgent assessment and intervention [10, 11].	1a	A
	34. A sudden decrease in drainage volume accompanied by abdominal distension, fever, and other symptoms should alert one to the potential blockage or displacement of the drainage tube, necessitating immediate examination and management [11].	1c	B
9. Patient health education	35. Prior to discharge, patient and caregiver education on self-care for the drainage tube is essential, including methods for emptying the drainage bag, recording drainage volume, skin care, activity precautions, and recognizing signs of infection [10, 11].	1a	A
	36. Inform patients of symptoms indicating infection related to the drainage tube: increased redness, warmth, pain at the puncture site, fever > 38 °C, cloudy or odorous drainage fluid, sudden increase or decrease in drainage volume; the presence of these symptoms should prompt immediate medical consultation [10, 17].	1a	A
	37. Patients with drainage tubes can shower normally after 48 h post-operation, but should keep the dressing dry or change it promptly after showering [6].	1b	B
	38. Establish a continuum of care model through phone follow-ups, mobile medical apps, or nursing clinics to provide ongoing guidance and monitoring for discharged patients with drainage tubes, thereby improving compliance and self-management [11].	1c	B
10. Indications for referral	39. Indications for urgent referral: Serious infections related to the drainage tube (signs of sepsis: fever > 38.5 °C, heart rate > 110 beats per minute, WBC > 12,000/µL), significant active bleeding, and drainage tube dislodgement with exposure or damage to internal organs [10, 11].	1a	A
	40. Indications for specialist consultation: Persistent abnormal drainage (excessive drainage still present after 7 days), continuous abnormal characteristics of drainage fluid (bile leak, pancreatic leak, lymphatic leak, etc.), drainage tube-related infections unresponsive to empirical treatment, complications requiring intervention or surgery [18, 20].	1c	B

## Discussion

### Standardization of drainage tube management as a crucial step in preventing SSI

Drainage tubes represent a commonly used auxiliary device after surgical procedures. The quality of its management directly affects the risk of surgical site infection (SSI). The updated 2024 version of the “Guidelines for SSI: Prevention and Treatment” issued by the UK NICE emphasizes the significant role of aseptic non-contact technology (ANTT) in the care of drainage tubes. It is recommended to cover the puncture point of the drainage tube with sterile dressings within 48 h after surgery [17].

A 2023 retrospective study from South Korea analyzed clinical data from 531 patients with retained drainage tubes to investigate the correlation between drainage tube characteristics and the occurrence of SSIs. It was found that the duration of drainage tube retention is a critical predictive factor for SSIs. Closed drainage systems, compared to open ones, significantly reduce the risk of infection [6]. However, the superiority of closed negative-pressure drainage over open systems may vary by surgical specialty and wound type; for example, certain low-risk clean wounds or specific plastic surgery procedures may not demonstrate the same magnitude of benefit [6]. The 2024 update of the SIS guidelines [18] further emphasizes standardized requirements for drainage tube selection and management, consistent with recommendations from WHO [9] and SHEA/IDSA [10].

### Evidence-based decision-making for the timing of drainage tube removal

The timing of drainage tube removal is a key factor affecting both SSIs and patient outcomes. A systematic review published in 2025 highlighted that identifying the optimal moment for the removal of surgical drainage tubes is essential for balancing the benefits of fluid drainage against the risks associated with prolonged device retention. Decision criteria based primarily on volume thresholds (30–50 mL/24 hours) and time-based standards are the two main approaches for determining removal timing [23].

A 2021 systematic review and meta-analysis by Shima et al., which included 11 studies on the timing of drainage tube removal following breast surgery, indicated that early removal led to a higher incidence of seroma compared to late removal (RR = 1.58), though there was no significant difference in the rate of SSIs (RR = 0.82). Early removal was associated with shorter hospital stays [24]. A 2024 analysis by Talwar et al. of the American NSQIP database suggested that prophylactic retention of drainage tubes might be linked to increased infection complications and extended hospital stays, indicating a need for stringent criteria for drainage tube placement [32].

It is important to note that the 30–50 mL/24-hour threshold for drain removal (Evidence 28) and the time-based recommendations (Evidence 29) are derived primarily from abdominal, breast, and orthopedic surgery literature and may not be directly applicable to all surgical specialties. For example, cardiac surgery, neurosurgery, and major vascular procedures may require specialty-specific removal criteria based on the nature of the surgical field and type of drainage output. Clinicians should adapt these general recommendations to their specific clinical context and specialty guidelines.

### Evidence-based recommendations for antimicrobial use

Regarding whether the use of prophylactic antimicrobial agents should be extended when a drainage tube is present, current evidence clearly supports not extending their use. In 2022, Pinchera and others published a review on SSI management, pointing out that a large systematic review confirmed that continuing the use of antimicrobial agents after 24 h post-surgery brings no benefit. Antimicrobial agents should be stopped at the time of wound closure, even if a drainage tube is present [26].

In 2024, Filardi et al. conducted a systematic review and meta-analysis of intraoperative wound irrigation systems, finding that diluted povidone-iodine irrigation reduced the risk of SSIs compared to saline irrigation (OR 0.573, 95% CI 0.321–0.953). However, routine antibiotic irrigation was not recommended [28]. These findings underscore the need to enhance evidence-based awareness among healthcare professionals to minimize unnecessary drug exposure and the risk of resistance.

### Application value of bundle measures in SSI prevention

In 2024, Crisafi et al. published findings from the ERAS Society for Cardiac Surgery, emphasizing the importance of multidisciplinary collaboration in the prevention of SSIs. They advocated for the incorporation of drainage tube management into perioperative bundled care protocols [20]. A meta-analysis involving 42 RCTs and 5,241 patients demonstrated that a bundled ERAS approach could reduce postoperative complications by 38% (RR 0.62, 95% CI 0.55–0.70), with SSIs specifically reduced by 27% (RR 0.73, 95% CI 0.56–0.95) [27].

The core strategy revolves around the principle of “restricted placement and early removal.” This includes stringent criteria for tube placement, dynamic assessment of drainage volume within 24–48 h post-operation with immediate removal upon meeting predefined criteria, the use of a sterile closed drainage system, and standardized skin disinfection. Continuous quality improvement is facilitated through daily checklists. This module, in coordination with other elements such as glucose control, temperature maintenance, and antibiotic management, forms an integral part of a standardized ERAS bundled care system.

### Evidence translation and practice: challenges and recommendations

The present study has several limitations: Of the 21 included articles, only three are high-quality RCTs, with the majority being expert consensus and systematic reviews, which constitute indirect evidence; optimal management strategies may vary between different types of surgeries and different types of drainage tubes; there is a scarcity of high-quality domestic research on drainage tube management, with most evidence derived from international studies; some of the evidence has short follow-up periods (≤ 30 days), lacking long-term outcome data. Additionally, the heterogeneity of included study designs (guidelines, systematic reviews, RCTs, cohort studies, and expert opinions) limits the ability to conduct a formal meta-analysis or to generate uniformly strong recommendations across all evidence statements. Some recommendations (e.g., Evidence 37 regarding showering with drainage tubes) are based on limited direct evidence and general infection prevention principles rather than drainage-specific comparative trials; such recommendations should be applied with caution and adapted to individual patient circumstances. The evidence summary presented should be regarded as a preliminary framework to guide clinical practice rather than definitive, universally applicable standards. All recommendations should be adapted to the specific surgical specialty, patient population, and institutional protocols.

### Conclusion

Future research directions should include conducting RCTs focused on drainage tube management for specific types of surgeries; developing intelligent decision-making systems for tube removal based on biochemical markers; evaluating the effectiveness of remote monitoring technologies in the management of patients discharged with drainage tubes; and performing cost-benefit analyses of bundled care measures for drainage tube management. Overall, this study provides a preliminary evidence-based framework for SSI prevention associated with drainage tubes, combining core recommendations from international guidelines with clinical practice in China. It is important to emphasize that the recommendations presented in this evidence synthesis should be adapted according to surgical specialty, patient-specific risk factors, and institutional protocols. Clinicians should interpret these general recommendations within their specific clinical context and consult specialty-specific guidelines where available. While this narrative evidence synthesis offers a comprehensive overview of current best practices, clinicians should recognize that the strength of individual recommendations varies, and implementation should be guided by the specific evidence grade and recommendation strength assigned to each statement. These recommendations should be interpreted in conjunction with procedure-specific guidelines and institutional protocols, as optimal drain management may vary among surgical specialties.

### Proposed clinical decision framework for drainage tube management

To facilitate the translation of the 40 evidence statements into bedside workflow, we propose a simplified clinical decision framework organized around three phases of drainage tube management (Table 7).

**Table 7:** Clinical decision framework for drainage tube management

Phase	Step	Key Action	Supporting Evidence	Notes
PHASE 1: PLACEMENT (Pre-operative to Intraoperative)	Step 1	Assess necessity based on surgical type and patient risk factors (diabetes, obesity, immunosuppression)	Evidence 1–4	Individualize decision; avoid routine prophylactic placement
	Step 2	Select a closed negative-pressure drainage system when indicated	Evidence 2	May vary by surgical specialty and wound type
	Step 3	Place via a separate puncture site (2–3 cm from the incision) using aseptic technique	Evidence 5, 9	Avoid placing a drain through the main incision
	Step 4	Position at the lowest point of the surgical field; confirm position and record type/location before closure	Evidence 6, 10	Ensure side holes are within the target drainage area
PHASE 2: MAINTENANCE (Post-operative Days 1–5)	Step 1	Perform a daily assessment of drain necessity	Evidence 22	Remove as soon as no longer needed
	Step 2	Maintain an aseptic non-touch technique during all handling	Evidence 12	Strict hand hygiene before and after
	Step 3	Monitor and document drainage fluid volume, color, consistency, and odor daily	Evidence 32–34	Watch for signs of infection, bile leak, or bleeding
	Step 4	Manage blockages: squeezing method, sterile saline flushing, or report to the physician	Evidence 16–20	Follow aseptic technique for flushing
	Step 5	Cover the puncture site with sterile dressings within 48 h post-surgery	Evidence 23	Assess the skin condition daily; change damp/loose dressings
PHASE 3: REMOVAL (When Criteria Met)	Step 1	Apply volume-based (< 30–50 mL/24 h) or time-based criteria by surgical specialty	Evidence 28–29	Specialty-specific adaptation required; see Evidence 31 for pancreatic/hepatobiliary surgery
	Step 2	Do not extend prophylactic antibiotics solely due to drain presence	Evidence 25	Stop antibiotics at wound closure
	Step 3	Provide patient education on self-care and warning signs before discharge	Evidence 35–38	Include showering instructions, dressing care, and when to seek help
	Step 4	Establish follow-up mechanisms for discharged patients	Evidence 38	Phone follow-ups, mobile apps, or nursing clinics

This framework is intended as a practical guide and should be adapted to institutional protocols and specialty-specific requirements

## Supplementary Information

Supplementary Material 1

Supplementary Material 2

## Data Availability

This is a narrative evidence synthesis of published literature; no primary patient data were collected or analyzed.
