# Supplementary material for: Summary of best evidence for drainage tube management to prevent surgical site infections
Source: BMC Surg. 2026 Jul 2;26:735. doi: 10.1186/s12893-026-03962-5 (PMC13726142; doi:10.1186/s12893-026-03962-5)
Supplement: Supplementary file 1 [file 12893_2026_3962_MOESM1_ESM.docx]

**Supplementary Material 1: Complete Database-Specific Search Strategies**

**PubMed (via NCBI)**

**Search date: October 28, 2025**

#1 "Surgical Wound Infection"[Mesh] OR "Surgical Site Infection"[Title/Abstract] OR "SSI"[Title/Abstract] OR "Postoperative Infection"[Title/Abstract] OR "Wound Infection, Surgical"[Title/Abstract]

#2 "Drainage"[Mesh] OR "Surgical Drain"[Title/Abstract] OR "Drain Management"[Title/Abstract] OR "Drain Care"[Title/Abstract] OR "Wound Drainage"[Title/Abstract] OR "Postoperative Drain"[Title/Abstract] OR "Drain Removal"[Title/Abstract] OR "Suction Drain"[Title/Abstract] OR "Closed Suction Drain"[Title/Abstract]

#3 "Infection Prevention"[Title/Abstract] OR "Infection Control"[Mesh] OR "Aseptic Technique"[Title/Abstract] OR "Antibiotic Prophylaxis"[Mesh] OR "Negative Pressure Wound Therapy"[Mesh]

#4 #1 AND #2

#5 #4 AND #3

#6 #4 OR #5

Filters: English, Chinese; Publication date: from inception to October 31, 2025.

**Embase (via Elsevier)**

**Search date: October 29, 2025**

#1 'surgical infection'/exp OR 'surgical site infection':ti,ab OR 'ssi':ti,ab OR 'postoperative infection':ti,ab OR 'wound infection':ti,ab

#2 'drainage'/exp OR 'surgical drain':ti,ab OR 'drain management':ti,ab OR 'drain care':ti,ab OR 'wound drainage':ti,ab OR 'postoperative drain':ti,ab OR 'drain removal':ti,ab OR 'suction drain':ti,ab OR 'closed suction drain':ti,ab

#3 'infection prevention':ti,ab OR 'infection control'/exp OR 'aseptic technique':ti,ab OR 'antibiotic prophylaxis'/exp OR 'negative pressure wound therapy'/exp

#4 #1 AND #2

#5 #4 AND #3

#6 #4 OR #5

Filters: English, Chinese; Publication date: from inception to October 31, 2025.

**Web of Science Core Collection (via Clarivate)**

**Search date: October 29, 2025**

#1 TS=("surgical site infection" OR "SSI" OR "postoperative infection" OR "surgical wound infection")

#2 TS=("surgical drain" OR "drain management" OR "drain care" OR "wound drainage" OR "postoperative drain" OR "drain removal" OR "suction drain" OR "closed suction drain")

#3 TS=("infection prevention" OR "infection control" OR "aseptic technique" OR "antibiotic prophylaxis" OR "negative pressure wound therapy")

#4 #1 AND #2

#5 #4 AND #3

#6 #4 OR #5

Filters: English, Chinese; Publication date: from inception to October 31, 2025.

**CINAHL (via EBSCOhost)**

**Search date: October 29, 2025**

S1 (MH "Surgical Wound Infection+") OR TI "surgical site infection" OR AB "surgical site infection" OR TI "SSI" OR AB "SSI" OR TI "postoperative infection" OR AB "postoperative infection"

S2 (MH "Drainage+") OR TI "surgical drain" OR AB "surgical drain" OR TI "drain management" OR AB "drain management" OR TI "drain care" OR AB "drain care" OR TI "wound drainage" OR AB "wound drainage"

S3 (MH "Infection Control+") OR TI "infection prevention" OR AB "infection prevention" OR TI "aseptic technique" OR AB "aseptic technique"

S4 S1 AND S2

S5 S4 AND S3

S6 S4 OR S5

Filters: English; Publication date: from inception to October 31, 2025.

**Cochrane Library (via Wiley)**

**Search date: October 30, 2025**

#1 MeSH descriptor: [Surgical Wound Infection] explode all trees

#2 ("surgical site infection" OR "SSI" OR "postoperative infection" OR "surgical wound infection"):ti,ab,kw

#3 MeSH descriptor: [Drainage] explode all trees

#4 ("surgical drain" OR "drain management" OR "drain care" OR "wound drainage" OR "postoperative drain" OR "drain removal"):ti,ab,kw

#5 MeSH descriptor: [Infection Control] explode all trees

#6 ("infection prevention" OR "aseptic technique" OR "antibiotic prophylaxis"):ti,ab,kw

#7 #1 OR #2

#8 #3 OR #4

#9 #5 OR #6

#10 #7 AND #8

#11 #10 AND #9

#12 #10 OR #11

Filters: Publication date: from inception to October 31, 2025.

**China National Knowledge Infrastructure (CNKI)**

**Search date: October 30, 2025**

检索词：((('手术部位感染' OR '术后感染' OR '切口感染') AND ('引流管' OR '引流' OR '引流管管理' OR '引流管护理')) AND ('感染预防' OR '无菌技术' OR '抗菌药物预防'))

检索范围：全部期刊

语言：中文、英文

发表时间：建库至2025年10月31日

**WanFang Database**

**Search date: October 30, 2025**

检索词：主题:(("手术部位感染" OR "术后感染") AND ("引流管" OR "引流") AND ("感染预防" OR "护理"))

检索范围：期刊论文

语言：中文、英文

发表时间：建库至2025年10月31日

**VIP Database (Weipu)**

**Search date: October 30, 2025**

检索式：M=(手术部位感染+术后感染+SSI)*(引流管+引流+引流管理)*(感染预防+无菌技术+护理)

检索范围：全部期刊

语言：中文

发表时间：建库至2025年10月31日

**China Biomedical Database (CBM)**

**Search date: October 31, 2025**

#1 "手术部位感染"[常用字段:智能] OR "术后感染"[常用字段:智能] OR "SSI"[常用字段:智能]

#2 "引流管"[常用字段:智能] OR "引流"[常用字段:智能] OR "引流管管理"[常用字段:智能]

#3 "感染预防"[常用字段:智能] OR "无菌技术"[常用字段:智能] OR "护理"[常用字段:智能]

#4 #1 AND #2

#5 #4 AND #3

#6 #4 OR #5

Filters: 中文、英文; 发表时间：建库至2025年10月31日

**ClinicalTrials.gov**

**Search date: October 31, 2025**

Condition: "surgical site infection" OR "SSI"

Intervention: "drain" OR "drainage" OR "drain management"

Status: All studies
