# Supplementary material for: Summary of best evidence for drainage tube management to prevent surgical site infections
Source: BMC Surg. 2026 Jul 2;26:735. doi: 10.1186/s12893-026-03962-5 (PMC13726142; doi:10.1186/s12893-026-03962-5)
Supplement: Supplementary file 2 [file 12893_2026_3962_MOESM2_ESM.docx]

**Supplementary Material 2: Evidence Matrix for Drainage Tube Management Recommendations**

| **Rec #** | **Recommendation Content** | **Evidence** | **Type** | **Level** | **Grade** | **Rationale** |
| --- | --- | --- | --- | --- | --- | --- |
| 1 | Placement based on clinical indications | [11,12] | Guideline | 1a | A | Multiple high-quality guidelines agree |
| 2 | Closed negative pressure preferred | [6] | Systematic review | 1a | A | Meta-analysis supports reduced SSI risk |
| 3 | Tube diameter and material selection | [11] | Expert consensus | 1c | B | Limited direct comparative evidence |
| 4 | Individualize based on surgical type | [10,17] | Guideline | 1b | B | Based on RCT and guideline consensus |
| 5 | Aseptic technique, check for twisting | [10,11,18] | Guideline | 1a | A | Strong guideline recommendation |
| 6 | Position at lowest point | [11,18,24] | Guideline + SR | 1b | A | Consistent across guidelines |
| 7 | Hepatic/biliary/pancreatic specifics | [11,18] | Guideline | 1b | A | Specialty-specific guidelines |
| 8 | Spinal surgery: avoid routine placement | [8] | Expert consensus | 1b | B | ERAS consensus recommendation |
| 9 | Separate puncture point 2-3cm from incision | [11] | Guideline | 1b | A | Strong evidence from guidelines |
| 10 | Confirm position before closure | [11,12] | Guideline | 1c | B | Expert consensus based |
| 11 | Non-absorbable sutures for fixation | [11] | Guideline | 1b | A | Standard surgical practice |
| 12 | Aseptic non-touch technique | [17] | Guideline | 1a | A | NICE guideline recommendation |
| 13 | Daily skin assessment | [10,17] | Guideline | 1a | A | Strong guideline consensus |
| 14 | Drainage bag management | [10,11] | Guideline | 1a | A | Multiple guidelines agree |
| 15 | Check for twists/compression daily | [6,32] | Cohort + Guideline | 1b | A | Consistent evidence |
| 16 | Blockage causes | [17,21] | Guideline + Evidence | 1b | A | Pathophysiology established |
| 17 | Squeezing method | [11] | Guideline | 1c | B | Expert consensus |
| 18 | Sterile saline flushing | [28] | Systematic review | 1a | B | Meta-analysis supported |
| 19 | Aseptic flushing procedure | [10,17] | Guideline | 1a | A | Strong guideline recommendation |
| 20 | Report if interventions fail | [21] | Evidence summary | 1b | A | Standard care protocol |
| 21 | Hand hygiene | [10] | Guideline | 1a | A | Core infection prevention |
| 22 | Daily assessment of necessity | [23,32] | SR + Cohort | 1a | A | Strong evidence for early removal |
| 23 | Sterile dressings within 48h | [17] | Guideline | 1a | A | NICE recommendation |
| 24 | 2% chlorhexidine alcohol | [1,2] | Guideline | 1b | A | CDC/WHO supported |
| 25 | No antibiotic extension for drain | [10] | Guideline | 1a | A | Strong guideline consensus |
| 26 | Prophylactic antibiotics within 60 min | [26] | Systematic review | 1a | A | Meta-analysis confirmed |
| 27 | Adjust based on culture results | [10,17] | Guideline | 1a | A | Standard practice |
| 28 | Volume-based removal <30-50mL/24h | [23] | Systematic review | 1a | B | Evidence supports criteria-based |
| 29 | Time-based removal by specialty | [12,24] | Guideline + SR | 1b | B | Varies by surgical type |
| 30 | Early removal does not increase SSI | [24,32] | SR + Cohort | 1a | A | Strong evidence |
| 31 | Special circumstances: amylase/bilirubin | [18] | Guideline | 1b | B | Specialty-specific |
| 32 | Daily documentation of drainage | [10] | Guideline | 1a | A | Standard monitoring |
| 33 | Abnormal drainage management | [10,11] | Guideline | 1a | A | Multiple guidelines |
| 34 | Sudden decrease alerts | [11] | Guideline | 1c | B | Expert consensus |
| 35 | Patient education before discharge | [10,11] | Guideline | 1a | A | Strong recommendation |
| 36 | Infection warning signs | [10,17] | Guideline | 1a | A | Multiple guidelines |
| 37 | Showering after 48h | [6] | Cohort | 1b | B | Limited direct evidence |
| 38 | Continuum of care model | [11] | Guideline | 1c | B | Expert consensus |
| 39 | Urgent referral indications | [10,11] | Guideline | 1a | A | Emergency criteria |
| 40 | Specialist consultation indications | [18,20] | Guideline | 1c | B | Specialty referral |
